# Factors Associated With Patients Not Receiving Oral Anticancer Drugs

**DOI:** 10.1001/jamanetworkopen.2022.36380

**Published:** 2022-10-13

**Authors:** Sahil D. Doshi, Morgan R. L. Lichtenstein, Melissa P. Beauchemin, Rohit Raghunathan, Shing Lee, Cynthia Law, Melissa K. Accordino, Elena B. Elkin, Jason D. Wright, Dawn L. Hershman

**Affiliations:** 1 Division of Medical Oncology, Department of Medicine, Memorial Sloan Kettering Cancer Center, New York, New York; 2 Divison of Hematology/Oncology, Department of Medicine, Columbia University Irving Medical Center, New York, New York; 3 Herbert Irving Comprehensive Cancer Center, Columbia University Irving Medical Center, New York, New York; 4 School of Nursing, Columbia University Irving Medical Center, New York, New York; 5 Mailman School of Public Health, Columbia University Irving Medical Center, New York, New York; 6 Division of Gynecologic Oncology, Department of Obstetrics and Gynecology, Columbia University Irving Medical Center, New York, New York

## Abstract

**Importance:**

Oral anticancer drugs (OACDs) are increasingly prescribed for cancer treatment and require significant coordination of care. Retrospective studies suggest that 10% to 20% of OACD prescriptions are never received by the patients, but the reasons behind this are poorly understood.

**Objectives:**

To estimate the rate of failure to receive OACD prescriptions among patients with cancer and to examine the underlying reasons for this failure.

**Design, Setting, and Participants:**

A prospective cohort study was conducted among patients with cancer who were prescribed a new OACD from January 1, 2018, to December 31, 2019, at an urban academic medical center. Data analysis was conducted between 2021 and 2022.

**Main Outcomes and Measures:**

Patient demographic, clinical, and insurance data and OACD delivery dates were collected. The reasons for a failure to receive a prescribed OACD within 3 months were confirmed by manual review of medical records and were classified into 7 categories: clinical deterioration, financial access, clinician-directed change in decision-making, patient-directed change in decision-making, transfer of care, loss to follow-up, and unknown or other. A multivariable random-effects model was developed to identify factors associated with failure to receive a prescribed OACD.

**Results:**

The cohort included 1024 patients (538 men [53%]; mean [SD] age, 66.2 [13.9] years; 463 non-Hispanic White patients [45%], 140 non-Hispanic Black patients [14%], and 300 Hispanic patients [29%]), representing 1197 new OACD prescriptions. Of the 1197 prescriptions, 158 (13%) were categorized as having not been received by the patient. The most common reason for the failure to receive a prescribed OACD was due to patient and clinician decision-making (73 of 158 [46%]), and 20 cases (13%) in which prescriptions were not received were associated with financial access issues. In multivariable analysis, patients with a nonmetastatic solid malignant neoplasm were significantly less likely to not receive their OACDs than those with a hematologic malignant neoplasm (odds ratio, 0.57 [95% CI, 0.33-1.00]; *P* = .048).

**Conclusions and Relevance:**

This cohort study of patients prescribed a new OACD found that 13% of prescriptions were not received. The failure to receive a prescribed OACD was most frequently due to a change in clinical decision-making or patient choice. Ultimately, the reasons for the failure to receive a prescribed OACD were multifactorial and may have been appropriate in some cases.

## Key Points

**Question:**

How many patients with cancer do not receive oral anticancer drug prescriptions, and what are the factors associated with this failure to receive these drugs?

**Findings:**

A cohort study of 1024 patients conducted at an urban academic medical center found that 13% of oral anticancer drug prescriptions were never received by the patient. The most common reason was because of patient and clinician decision-making; 13% of cases of failure to receive an oral anticancer drug prescription were directly associated with financial access issues.

**Meaning:**

This study suggests that the failure to receive oral anticancer drugs is infrequent and that the reasons are multifactorial.

## Teaser

This cohort study estimates the rate of failure to receive oral anticancer drug prescriptions among patients with cancer and examines the reasons associated with this failure.

## Introduction

Oral anticancer drugs (OACDs) have been available for decades, but there has been an increase in US Food and Drug Administration (FDA) approvals for OACDs in recent years. In 2020, 67% of newly approved cancer agents were OACDs. Oral anticancer drugs are often a convenient alternative to parenteral therapies and can reduce the frequency of travel and appointments.^1,2^ However, like other novel therapies, OACDs are often expensive, with high out-of-pocket costs, which could lead to delays in the receipt of medication and the failure to initiate treatment.^3,4^ There has been increasing focus on understanding and improving OACD adherence.^5^

The literature on OACD access is limited, to our knowledge. A 2011 retrospective study found that 10% of patients prescribed a new OACD abandoned the prescription and did not fill a substitute prescription in the subsequent 90 days.^6^ A second retrospective study found an 18% abandonment rate for OACDs, defined as a failure to retrieve a prescription from the pharmacy within 90 days, which was associated with higher out-of-pocket drug costs.^7^ Several factors have been proposed as barriers to initiation of OACDs, including patient beliefs regarding treatment effectiveness and adverse effects, as well as the financial burden, including copayments and insurance approval.^5,7,8^ However, these studies have not been able to directly assess the importance of these factors because they have relied largely on insurance and pharmacy claims data. We therefore conducted a prospective study among oncology patients to improve our understanding of patients’ failure to receive OACDs and the demographic and clinical factors associated with this failure to receive OACDs.

## Methods

### Study Population

In this prospective cohort study, all adult oncology patients (aged >18 years) at the Columbia University Irving Medical Center (CUIMC) in New York, New York, who received a new OACD prescription from January 1, 2018, to December 31, 2019, were eligible for inclusion. The OACDs were prescribed by oncologists at CUIMC and processed by a specialty pharmacy. Patients previously prescribed the same drug as noted in the electronic health record (EHR) or who received the OACD as part of a clinical trial were excluded. Patients receiving endocrine therapy alone for breast cancer were excluded. Patients could be included multiple times if they had multiple new OACD prescriptions. This study was approved by the CUIMC institutional review board. All included study participants had previously provided written informed consent to allow for the review of EHRs for research purposes. This study followed the Strengthening the Reporting of Observational Studies in Epidemiology (STROBE) reporting guideline for observational studies.

### Data Collection

Demographic information (age, sex, race and ethnicity, tumor type, and insurance) was collected at the patient level, while prescription-specific information (prior authorization [PA], drug class, and years since FDA approval) was collected at the prescription level. Race and ethnicity were collected from patient-reported information and classified as non-Hispanic White, non-Hispanic Black, Hispanic of any race, and other (including non-Hispanic Asian, Pacific Islander, and Alaska Native).

For data collection, a paper-based case report form was created for each patient, and the clinic nurses completed the forms over time until the delivery of the prescription. The information collected included the OACD prescription name, the date of the prescription, and the date of the delivery of the OACD or the failure date (the date when it was noted that the patient had not received the OACD). About one-third of the way into the study, a hospital-based specialty pharmacy was implemented at CUIMC to facilitate the procurement and delivery of the OACD. All OACD prescriptions from the oncology clinics were subsequently submitted directly to the hospital-based specialty pharmacy, which collected all of the information previously collected on the case report forms electronically.

Information about the reasons for the failure to receive a prescribed OACD within a 3-month period from time of prescription during the study was initially obtained from a database derived from the case report forms and the hospital-based specialty pharmacy. If the reasons were not clear, then we retrospectively performed a manual review of the outpatient oncology encounter notes and pharmacy notes. A group consensus was reached to categorize the reasons for a failure to receive a prescribed OACD into 7 distinct categories to highlight key reasons and reduce redundancy. These reasons for a failure to receive a prescribed OACD were categorized as (1) clinical deterioration (death or hospice), (2) financial access (issues with insurance and affordability), (3) clinician-directed change in decision-making (oncologists opting for a different drug or clinical trial), (4) patient-directed change (patients pursuing different treatments), (5) transfer of care (patients pursuing care at another institution), (6) loss to follow-up, and (7) unknown or other (used when the reason for failure to receive a prescribed OACD was not clear in the EHR). Any patient who had missing data for the primary outcome or covariates was excluded from the study.

### Statistical Analysis

Descriptive statistics were used to describe patient and prescription-level characteristics. Binary and categorical variables, including baseline characteristics and the failure to receive prescribed OACDs, were summarized as frequencies and proportions. The χ^2^ test was used to analyze associations between patient sociodemographic, prescription, and process factors. To evaluate the association of drug class, PA, and time since FDA approval with the primary outcome of failure to receive prescribed OACDs, univariate and multivariate logistic mixed-effects models were performed. A patient-specific random effect was included to account for repeated observations among patients. The multivariate model included other fixed-effects variables to adjust for age, race and ethnicity, and tumor stage and type. Insurance was not included because of its collinearity with age and race and ethnicity. The χ^2^ test of independence was used to assess the collinearity between the adjusted variables to prevent an overfitted model. To select between surrogate variables, the Akaike Information Criterion was used to assess the quality of fit for each model. All *P* values were from 2-sided tests, and results were deemed statistically significant at *P* < .05. A sensitivity analysis was also performed to examine only the first observation for patients with multiple new OACD prescriptions during the study period to exclude those with prior exposure to the OACD process. All analyses were conducted using R, version 3.6.1 (R Group for Statistical Computing). The packages used for this analysis included lme4, stats, and tableone.

## Results

The study cohort included 1024 patients (538 men [53%]; mean [SD] age, 66.2 [13.9] years; 463 non-Hispanic White patients [45%], 140 non-Hispanic Black patients [14%], and 300 Hispanic patients [29%]) with 1197 new prescriptions (Table 1). Of the 1024 patients, 154 (15%) had 2 or more prescriptions during the study. Of 1024 patients, 602 (59%) had Medicare, 160 (16%) had Medicaid only, and 262 (25%) had commercial insurance only. Most OACDs (674 of 1197 [56%]) were targeted therapies, and 447 of the 1197 OACDs (37%) were approved by the FDA within 5 years of the prescription date.

**Table 1.** Demographic and Clinical Characteristics of Patients and Prescription Drugs

Category	Participants, No. (%)
**Patient level (N = 1024)**	
Age, y	
<50	124 (12)
50-59	159 (16)
60-69	282 (27)
>69	459 (45)
Sex	
Female	486 (47)
Male	538 (53)
Race and ethnicity	
Hispanic (of any race)	300 (29)
Non-Hispanic Black	140 (14)
Non-Hispanic White	463 (45)
Other^a^	121 (12)
Tumor type	
Hematologic	254 (25)
Solid metastatic	510 (50)
Solid nonmetastatic	260 (25)
Insurance type	
Any Medicare	602 (59)
Commercial only	262 (25)
Medicaid only	160 (16)
**Prescription level (N = 1197)**	
Prior authorization requirement	
Not required	359 (30)
Required	838 (70)
Drug class	
Chemotherapy	351 (29)
Hormonal	172 (14)
Targeted	674 (56)
FDA drug approval period, y	
>5	750 (63)
≤5	447 (37)

Abbreviation: FDA, US Food and Drug Administration.

^a^ Included non-Hispanic Asian, Pacific Islander, and Alaska Native.

Of the 1197 prescriptions, 158 (13%) were categorized as having not been received by the patient. Univariate analysis showed that the likelihood of this failure to receive prescribed OACDs was higher for targeted drugs compared with chemotherapy (odds ratio [OR], 1.57 [95% CI, 1.01-2.43]; *P* = .04) and drugs approved for 5 years or less compared with those approved for more than 5 years (OR, 1.67 [95% CI, 1.15-2.41]; *P* = .007). However, these odds were not statistically significant after adjusting for demographic and clinical covariates (targeted drugs vs chemotherapy: OR, 1.36 [95% CI, 0.84-2.20; *P* = .21; drugs approved for ≤5 years vs >5 years: OR, 1.36 [95% CI, 0.84-2.20]; *P* = .21; Table 2). In multivariable analysis, patients with a nonmetastatic solid malignant neoplasm were significantly less likely to not receive OACDs compared with those with a hematologic malignant neoplasm (OR, 0.57 [95% CI, 0.33-1.00]; *P* = .048). Additionally, there was a trend toward significance for failure to receive prescribed OACDs to be less likely in cases in which a PA was initiated (OR, 0.69 [95% CI, 0.47-1.01]; *P* = .06). A sensitivity analysis evaluating only the first OACD prescription for all patients in our study demonstrated a nearly identical abandonment rate of 14% (139 of 1024 prescriptions). In multivariable analysis, the association between failure to receive prescribed OACDs and tumor type as well as the association between failure to receive prescribed OACDs and PA had a similar effect size but wider 95% CIs and were no longer statistically significant.

**Table 2.** Multivariable Analysis of Factors Associated With the Failure to Receive Oral Anticancer Drugs

Factor	No. (%) (N = 158)	OR (95% CI)	*P* value
Age (continuous)			
1-y Increments	NA	1.00 (0.99-1.02)	.69
Race and ethnicity			
Non-Hispanic White	80 (51)	[Reference]	NA
Hispanic (of any race)	46 (29)	0.92 (0.61-1.41)	.71
Non-Hispanic Black	15 (9)	0.61 (0.33-1.14)	.11
Other^a^	17 (11)	0.84 (0.46-1.53)	.57
Tumor type			
Hematologic	49 (31)	[Reference]	NA
Solid metastatic	81 (51)	0.71 (0.46-1.10)	.13
Solid nonmetastatic	28 (18)	0.57 (0.33-1.00)	.048
Prior authorization requirement			
Not required	55 (35)	[Reference]	NA
Required	103 (65)	0.69 (0.47-1.01)	.06
Drug class			
Chemotherapy	38 (24)	[Reference]	NA
Hormonal	15 (10)	0.90 (0.44-1.82)	.77
Targeted	105 (66)	1.36 (0.84-2.20)	.21
FDA drug approval period, y			
>5	83 (53)	[Reference]	NA
≤5	75 (47)	1.36 (0.84-2.20)	.21

Abbreviations: FDA, US Food and Drug Administration; NA, not applicable; OR, odds ratio.

^a^ Included non-Hispanic Asian, Pacific Islander, and Alaska Native.

The 158 cases in which patients did not receive a prescribed OACD were categorized into 7 reasons for failure to receive. Overall, 47 (30%) were associated with clinician-directed change in decision-making, 26 (16%) with patient-directed change, 29 (18%) with clinical deterioration, 20 (13%) with financial access, 19 (12%) with transfer of care, and 9 (6%) with loss to follow-up (Figure).

**Figure.**
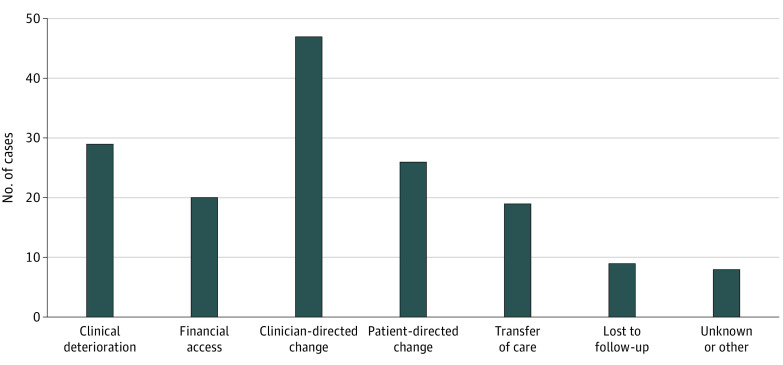
Categories of Reasons for the Failure to Receive Oral Anticancer Drugs

## Discussion

In a prospective evaluation of patients with cancer prescribed new OACDs, we found that 13% of prescriptions were never received and that the failure to receive OACD prescriptions was less likely among patients with nonmetastatic solid cancers. The most common reason for not receiving OACD prescritions was a change in patient or clinician decision-making (46%), whereas 13% of cases in which patients did not receive a prescribed OACD were associated with financial access issues.

Our findings of a 13% abandonment rate are consistent with other retrospective studies that have found rates ranging from 10% to 18% for OACD noninitiation or abandonment.^6,7^ Studies limited to hormonal treatment for breast cancer, often obtained through nonspecialty pharmacies, have demonstrated a wider noninitiation rate (between 13% and 19% from self-reported results to between 14% and 30% from EHR and pharmacy records).^8,9,10,11,12^

Our study found that the failure to receive OACD prescriptions was less likely among patients with nonmetastatic solid cancers. It appears that adjuvant therapy was more likely to be received compared with palliative treatment, which may reflect more frequent changes in clinician or patient decision-making or clinical deterioration among patients with advanced cancer. We also found that the failure to receive OACD prescriptions was less likely when a PA was initiated, which was an unexpected finding. Although this PA requirement could lead to a delay in the receipt of an OACD, it is possible that the patients who initiated the PA process had more financial assistance to complete the process.

The increased availability of OACDs raises new financial challenges in cancer care delivery. Our study found that only 13% of the cases in which patients did not receive a prescribed OACD were due to financial access issues, although the potential influence of financial factors on clinician and patient decision-making remains unclear.

### Limitations

This study has some limitations. Although this study provides a more granular description of the reasons for the failure to receive OACD prescriptions, our analysis is limited by the data available from pharmacy databases and the EHR at a single institution. Second, we recorded only the primary reason for a failure to receive an OACD prescription, and although this primary reason was determined after a rigorous review process of the information available in the EHR, there may have been multiple reasons why patients did not receive their OACD prescriptions. Third, we were not able to access information on cost, including Medicare Part D coverage information and out-of-pocket requirements, to see whether cost was associated with the failure to receive an OACD prescription, as seen in other studies.

## Conclusions

In this prospective cohort study of participants prescribed OACDs, we found that most oncology patients prescribed new OACDs received their drug, but 1 in 8 prescriptions were never delivered. Although the failure to receive OACD prescriptions is infrequent and may often be associated with clinician or patient decision-making, financial and educational interventions may be appropriate to ensure treatment access. Future studies may benefit from direct collection of patient- and clinician-reported information to better understand the reasons for the failure to receive prescription OACDs.
